# Modeling multiple sea level rise stresses reveals up to twice the land at risk compared to strictly passive flooding methods

**DOI:** 10.1038/s41598-018-32658-x

**Published:** 2018-09-27

**Authors:** Tiffany R. Anderson, Charles H. Fletcher, Matthew M. Barbee, Bradley M. Romine, Sam Lemmo, Jade M.S. Delevaux

**Affiliations:** 1 University of Hawaiʻi at Mānoa, Department of Geology and Geophysics, School of Ocean and Earth Science and Technology, Honolulu, HI 96822 USA; 2 Geography Research Unit, University of Oulu, P.O. Box 8000, Oulu, 90014 Finland; 3 University of Hawaiʻi Sea Grant College Program, Honolulu, HI 96822 USA; 4 Hawaiʻi Department of Land and Natural Resources, Office of Conservation and Coastal Lands, Honolulu, HI 96813 USA

**Keywords:** Environmental impact, Climate-change adaptation, Climate-change impacts, Natural hazards

## Abstract

Planning community resilience to sea level rise (SLR) requires information about where, when, and how SLR hazards will impact the coastal zone. We augment passive flood mapping (the so-called “bathtub” approach) by simulating physical processes posing recurrent threats to coastal infrastructure, communities, and ecosystems in Hawai‘i (including tidally-forced direct marine and groundwater flooding, seasonal wave inundation, and chronic coastal erosion). We find that the “bathtub” approach, alone, ignores 35–54 percent of the total land area exposed to one or more of these hazards, depending on location and SLR scenario. We conclude that modeling dynamic processes, including waves and erosion, is essential to robust SLR vulnerability assessment. Results also indicate that as sea level rises, coastal lands are exposed to higher flood depths and water velocities. The prevalence of low-lying coastal plains leads to a rapid increase in land exposure to hazards when sea level exceeds a critical elevation of ~0.3 or 0.6 m, depending on location. At ~1 m of SLR, land that is roughly seven times the total modern beach area is exposed to one or more hazards. Projected increases in extent, magnitude, and rate of persistent SLR impacts suggest an urgency to engage in long-term planning immediately.

## Introduction

Global mean sea level rise (SLR) is accelerating^1^. Satellite altimetry from 1993 to 2015 indicates it rose nearly three times faster (~3 mm/yr) than during the previous century (~1.2 mm/yr)^2,3^. Depending on the trajectory of global greenhouse gas emissions, SLR may reach 1 m or more by the end of the century^4^, especially if the contribution from Antarctic ice melt has been under-predicted^5^.

In Hawai‘i, local long-term SLR is presently mild (1.44 ± 0.21 mm/yr^6^). But local SLR could reach 8 mm/yr during the second half of this century, which would exceed global mean SLR. This is due to gravitational redistribution of meltwater leading to higher sea levels in the equatorial Pacific (near Hawai‘i), relative to other oceanic locations^7^. Additionally, the 10 to 20 cm of SLR expected no later than 2050, will more than double the frequency of extreme water-level events in the Tropics, where Hawai‘i is located^8^. The main Hawaiian Islands, a developed high island chain with low-lying coastal plains (typically <3 m above sea level), might serve as a model for other Pacific islands and island nations for understanding and adapting to SLR in the Tropics.

**Figure 1:**
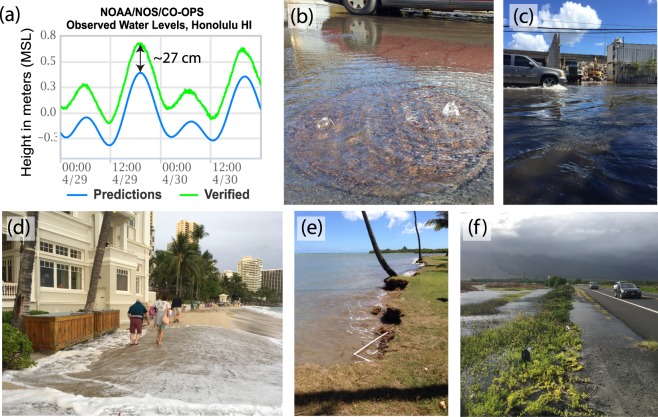
Between April–August, 2017, an episodic sea level anomaly, combined with long-term SLR triggered (**a**) up to ~27 cm of difference between predicted tides and observed water levels^66^. This heightened water level led to (**b**) salt water intruding storm drains, (**c**) urban flooding, (**d**) wave inundation (Waikīkī, O‘ahu), (**e**) episodic erosion (exposing a watering system), and (**f**) roadway flooding near wetlands due to groundwater inundation. (Photos: Hawai‘i Sea Grant King Tides Project, http://ccsr.seagrant.soest.hawaii.edu/king-tides).

Because coastal zones are densely populated and have high population growth rates, it is expected that increasing numbers of people will be exposed to SLR hazards in the future^9^. When mean water level rises, it leads to: marine flooding^10^; drainage problems, due to a rise in the groundwater table^11,12^, as well as saltwater blockage of drains and other conduits connected to the ocean^13^; saltwater intrusion which changes coastal ecosystems and aquifers^14^; changes in wave dynamics as water depths increase over reefs and other coastal structures^15^, which in turn can lead to increased wave power at the shore^16^; and increased beach erosion^17,18^. These impacts are further exacerbated when higher mean sea level (related to climate change) combines with episodic rises in sea level caused by other processes such as storm surge^19,20^, tides^21^, eddies^22^, wave events^23^, and regional climate indices (e.g. Pacific Decadal Oscillation and Southern Oscillation Index^24^, El Niño – Southern Oscillation^25^). For example, a recent high water anomaly April–August, 2017, coupled with seasonal wave events, led to wave inundation, episodic erosion, groundwater flooding, and saltwater blockage of drainage infrastructure at vulnerable sites in Hawai‘i (Fig. 1). This event provided residents a glimpse of flood conditions under higher mean sea levels.

In general, flooding at low-lying elevations in Hawai‘i is observed annually, during highest astronomical tides, whenever background water level anomalies exceed just a few tens of centimeters^22^. The frequency of these events has also increased due to the upward trend of sea level^22^. Observed worldwide, these floods, while individually mild, can cumulatively cause greater damage than a single extreme event^26^. Therefore, it is important for communities to plan for the effects of SLR, especially those that occur frequently enough to impart chronic effects on coastal zones.

Before planning can begin, it is necessary to identify coastal areas that are vulnerable to SLR. This is challenging because sea level-related hazards vary in their intensity and spatial distribution across landscapes due to local topography, bathymetry, and wave exposure. To produce spatially-explicit estimates, localized modeling of complex coastal processes is needed. However, available models typically require large, detailed datasets and are computationally intensive^27,28^. So, it is common for local jurisdictions to develop SLR adaptation guidance on the basis of either no modeling; or on a simple passive (or “bathtub”) terrain flood model^29^. The passive flood model is a projection of a flood surface onto a digital elevation model (DEM). The flood surface, in its simplest form, is a horizontal plane of pre-determined height or elevation (e.g. SLR). It is easy to implement and provides the requisite spatial specificity.

However, passive flood modeling does not provide a comprehensive picture of SLR hazards because it ignores dynamic processes such as seasonal waves, storm surge, and erosion, which have been shown to have a considerable impact on coastal communities^18,30–34^. Despite the documented need for more comprehensive impact modeling, only a limited number of studies have modeled multiple SLR-related processes at a spatial scale appropriate for local planning^28,33^.

To address this gap and inform local planning, we model recurring coastal processes (e.g. waves, tidally forced groundwater inundation) to comprehensively quantify the projected land area exposed to sea level-related hazards. We examine how land exposure changes, as a function of SLR, and investigate the relative importance of modeling each coastal process, in terms of the overall exposure. To identify hazards specific to each area, we take a place-based approach and use models that capture SLR impacts at the local level. We use a combination of numerical and empirical models to estimate the chronic impacts of SLR under the RCP 8.5 emissions pathway on all low-lying coasts of three of the most populated Hawaiian Islands: O‘ahu, Kaua‘i, and Maui.

## Approach and Models

Modeling is performed under four higher sea level scenarios – corresponding to the upper limit of the likely range (83rd percentile) for years 2030, 2050, 2075, and 2100, as set forth in IPCC AR5 RCP8.5 – and a baseline scenario (2015) representing present-day conditions (Table 1; see Supplementary Material for more detail). We model prevalent environmental processes that, under SLR, pose direct and frequent threats to Hawai‘i coastal infrastructure, communities, and ecosystems. These are: marine overland flooding of low-lying areas; groundwater flooding; storm drain backflow from the ocean; wave inundation due to seasonal (non-storm) wave run-up and overwash; and erosion of sandy shorelines. The following sub-sections explain why each physical process was selected and a brief description of the method used to model them. Detailed information on models and data are included in the Methods section.

**Table 1:** IPCC AR5 RCP 8.5 global mean sea level projections for selected future years, relative to 1986–2005.

Year	Lower (m) (18th percentile)	Median (m) (50th percentile)	Upper (m) (83rd percentile)
2015 (baseline)	0.05	0.06	0.08
2030	0.10	0.13	0.17
2050	0.19	0.25	0.32
2075	0.34	0.46	0.60
2100	0.53	0.74	0.98

Future SLR scenarios in this study correspond to the upper limit of the likely range (right column).

### Passive flooding at high tide

Low elevation coastal plains and shallow groundwater tables^35^ are prevalent in Hawai‘i. Low coastal lands are vulnerable to flooding from multiple sources, including: a rising groundwater table^11,35^; seawater flowing from the ocean through storm drains and out into urban areas (storm drain backflow from the ocean); and seawater flowing directly across the shoreline into lands that lie below the water level. Our methodology does not distinguish between water table flooding and inland storm drain backflow; hence we refer to them collectively as “groundwater flooding/storm drain backflow.” Direct flow of seawater across the shoreline we refer to as “marine overland flooding”.

We follow the method in Cooper *et al*.^10,36^ to map low-lying areas exposed to flooding under SLR. First, a temporally static yet spatially varying mean higher high water (MHHW) surface (spatially varies to adjust for small differences in MHHW between local tide gauges) is created. Future tidal surfaces are then created by increasing the water level above the MHHW surface by the projected amount of SLR. Flooding during high tide is modeled by subtracting the tidal surface model from a DEM (the DEM is described in Methods) using ArcGIS^37^. If, from a map view, the boundary of a mapped flooded area is connected to the ocean, that area is identified as “marine overland flooding”. If the boundary of a mapped flooded area is unconnected to the ocean (land-locked), we assume that it represents either groundwater flooding or inland storm drain backflow from the ocean.

In our method, we assume that the groundwater level is equal to the predicted tidal heights. Habel *et al*.^38^ found that three-dimensional groundwater modeling at high tide in Hawai‘i was well represented by the passive MHHW surface. Monitored well sites in Hawai‘i show that during high tide, the groundwater level near the coast is similar to the tidal elevation; while farther inland, the contribution from tides is reduced, but is compensated by an elevated mean groundwater level^35^, which results in a groundwater surface that mimics the high tidal elevation. We also assume that groundwater levels will increase at the same rate that SLR is occurring because we assume that the water table is perched on the salt water.

### Wave inundation

Storm surge associated with hurricanes can cause significant damage to island communities. However, it is rare in Hawai‘i. More common are swells, series of long-period waves that are generated from distant storms, which impact Hawaiian shores on a seasonal basis^39,40^. As swells dissipate across reefs, they lead to wave set-up (increased water elevation) and an increase in infragravity waves (longer period waves), which contribute to wave run-up and overwash^41–43^. Since any rise in mean sea level will increase the mean water level over the reef, the response of wave inundation to SLR is non-linear. Accordingly, we use a numerical model to capture changes in wave dynamics as sea level rises, and model the dominant annual wave climate at all shores, even if the waves are shorter period wind waves.

We model the seasonal high wave event for each location and for each SLR scenario with the non-hydrostatic XBeach model^44–46^, which is widely used in assessing vulnerability to SLR in both reef and non-reef environments^28,47^. The 1-dimensional (1D) model was selected because the more robust 2-dimensional (2D) model requires a large computation time in non-hydrostatic mode, making it infeasible for large-scale application. Modeling is performed along shore-normal profiles spaced 20 m alongshore. Modeled parameters (depth, velocity) are interpolated to create 2D grids. Any parameters at grid locations with velocity <0.1 m/s or depth <0.1 m are omitted to ignore static, inland water that does not result from waves, and to remove thin veneers of water excursion. This last step also helps to distinguish between active wave-induced floodwater and standing water (passive marine overland flooding or groundwater/drainage flooding). The resulting wave inundation is the horizontal extent of the modeled wash of the waves. Data and boundary conditions such as the DEM, wave forcing, and bottom friction values are described in the Methods section.

### Chronic coastal erosion

Coastal erosion is prevalent in the Hawaiian Islands. A previous study estimates that roughly 70% of beaches on the islands of Kaua‘i, Maui, and O‘ahu are retreating, and 21.5 km (11%) of those beaches have been completely lost to chronic erosion and shoreline hardening over the 80-year study period^48^. Larger SLR rates have been correlated with increased rates of shoreline retreat in Hawai‘i^49^, and future SLR is likely to exacerbate retreat rates^17^.

To estimate future erosion hazards, we use the probabilistic method presented by Anderson *et al*.^17^, which combines the long-term historical shoreline change rates with a geometric Bruun-type model to adjust for future SLR (in excess of historical SLR). The equation for the projected vegetation line follows from the shoreline change equation in Anderson *et al*.^17^, and is

1 $${y}_{veg}(t)={y}_{veg}({t}_{0})+r(t-{t}_{0})-({S}_{f}-{S}_{hist})/\tan \,\beta $$

where *y*_*veg*_(*t*) is the vegetation position at future time *t*, *y*_*veg*_(*t*_0_) is the vegetation position at initial time *t*_0_, *r* is the historical shoreline change trend, (*S*_*f*_ − *S*_*hist*_) is the difference between the IPCC projected sea level and the extrapolated Hawai‘i sea level at time *t*, and tan*β* is the slope of the submerged portion of the active beach profile. In this equation, negative values indicate retreat. Erosion hazard areas are created in ArcGIS by connecting an erosion hazard line (determined from the projected vegetation) with the present-day shoreline (zero elevation, mean sea level (MSL) contour), to represent land at risk of future erosion.

It is important to note that the erosion model differs from the passive and wave models, in that it depends on the time it takes for sea level to reach a pre-determined elevation. By contrast, the passive and wave models can be applied to a higher sea level, regardless of when that higher sea level occurs.

## Results

From the above-described modeling, TetraTech Inc. produced maps depicting the spatial extent of land exposed to modeled hazards. These maps, covering the islands of Kaua‘i, O‘ahu, and Maui, are available for viewing and download through the Pacific Islands Ocean Observing System^50^ (See Supplemental Material for technical differences between the online maps, and the spatial layers used in this study). Here, we present a subset of these map areas to illustrate the spatial distribution of coastal lands exposed to coastal hazards, under several SLR scenarios, for one location on O‘ahu. We then present the quantitative results: initially, we show land area exposed to individual hazards and the cumulative (overall) exposure as a function of SLR; thereafter, we present rates of change in exposure area with respect to SLR; lastly, we display the contribution of each modeled physical process to the overall exposure.

### Mapping exposure of coastal communities to coastal hazards

**Figure 2:**
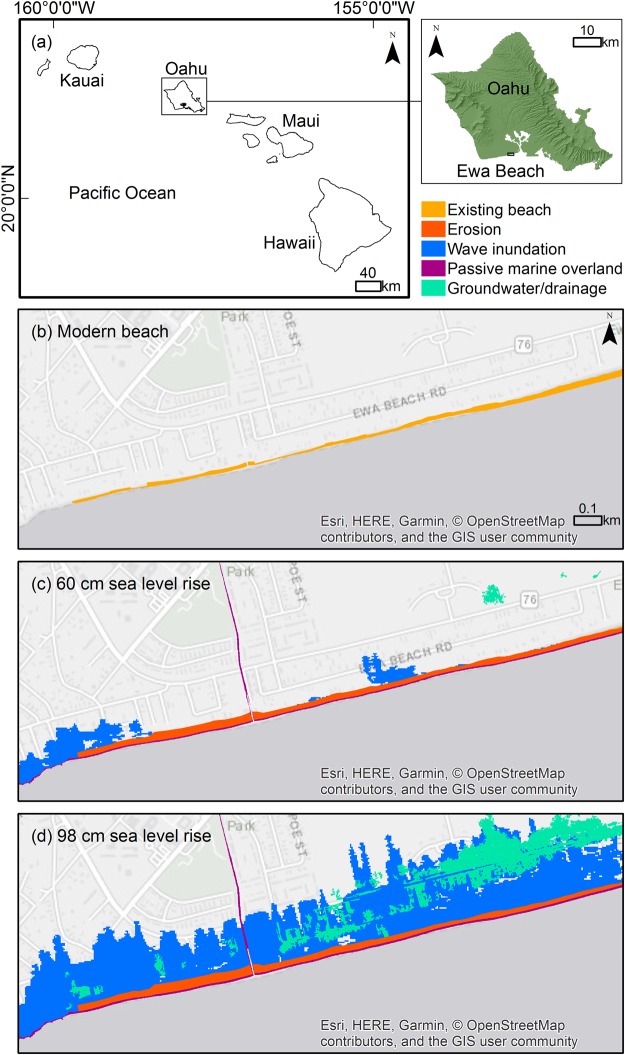
Modeled exposure to sea level-related hazards at ‘Ewa Beach, O‘ahu, show the localized spatial distribution of lands exposed to passive marine overland flooding at high tide (maroon), passive groundwater flooding/drainage backflow from the ocean at high tide (green), seasonal wave inundation (dark blue), and chronic erosion (red). The existing beach area is shown in orange; (**a**) location map; (**b**) present-day beach; (**c**) 0.60 m SLR scenario; (**d**) 0.98 m SLR scenario. (The cartography in the OpenStreetMap map tiles is licensed under CC BY-SA. The license terms can be found at: www.openstreetmap.org/copyright).

To illustrate the spatial distribution of lands exposed to hazards, Fig. 2 shows the suburban community of ‘Ewa Beach, on the island of O‘ahu, under three sea level scenarios. The area consists mainly of carbonate sand-rich coastal plains, fronted by low dunes near the shore; an exposed limestone shelf (Pleistocene) exists to the left of the existing beach and also under nearby homes. This figure shows only part of the larger ‘Ewa area, where population has more than doubled between the years 2000 and 2010^51^, and many homes are being renovated and expanded.

Passive marine overland flooding (maroon; marine water that has come directly across the shoreline at MHHW) is minimal at ‘Ewa Beach, even in the 0.98 m SLR scenario. By contrast, passive groundwater/storm drain flooding (green), representing both outcropping water table and salt-water intrusion of drainage infrastructure, is minimal, until the 0.98 m scenario, in which large green areas indicate flooding of the presently-shallow water table. Wave inundation (dark blue) is the result of modeling the annual high wave event. The ‘Ewa Beach area is most prone to long-period southern swells during the summer months (June–August). Wave parameters for ‘Ewa Beach, derived from an extreme value statistical analysis of offshore wave data (see Methods for details), are: 1.8 m significant wave height, 16 second peak period, and 181°N dominant direction (waves approaching from the south). Waves begin to overtop the limestone bench (left side of image) and a portion of the beach berm (middle of image) at 0.60 m of SLR. A considerably larger area is exposed to wave inundation for the 0.98 m SLR scenario. Coastal erosion (red) has the largest relative impact on land area during lesser amounts of SLR (0.17 and 0.32 m SLR scenarios), but is milder than the other hazards at higher levels of SLR. However, due to the density of oceanfront homes, small amounts of erosion will have a substantial impact to the existing beach. Most erosion occurs at the left side of the beach, adjacent to the limestone bench, where sand dunes have the lowest elevations, due to the density of oceanfront homes. For all hazards, the spatial distribution shows great dependence on local topography and geology.

### Land area exposed to hazards at higher sea levels

**Figure 3:**
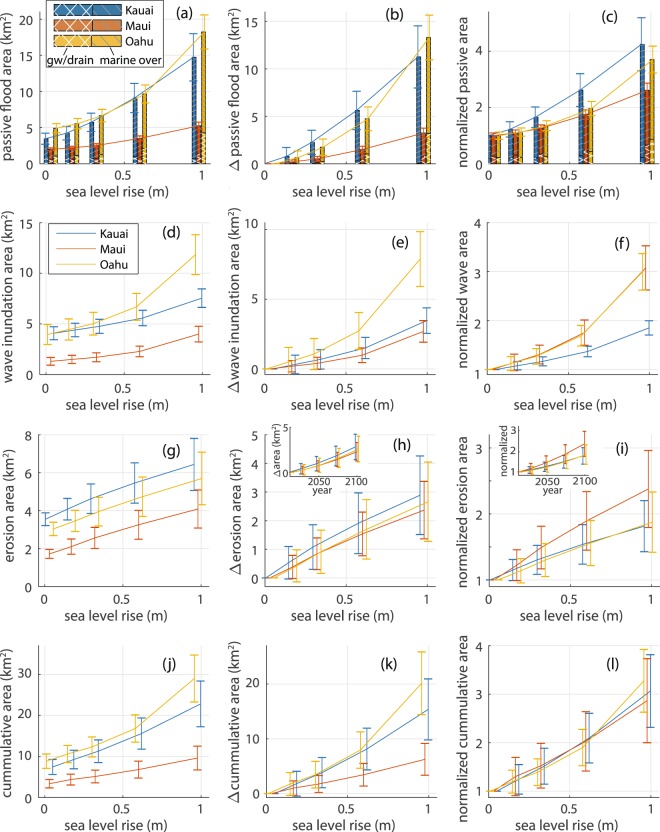
Quantification of land area exposed to each hazard due to increased SLR is shown for the islands of Kaua‘i (blue), Maui (yellow), and O‘ahu (red) for the SLR scenarios: present-day (0.08), 0.17, 0.32, 0.60, and 0.98 m. Plots show: (left column) exposed areas; (middle column) change in exposed area relative to the present-day scenario (future area minus present-day area); and (right column) normalized area (future area divided by present-day area). Error bars represent standard errors. Land areas exposed to passive flooding (**a**–**c**) are shown as stacked histograms, where the height of each bar represents the total (passive marine overland + groundwater/drainage) land area exposed, with marine overland contributions shown with gray single-hatching and groundwater/storm drain contributions shown with white cross-hatching. Wave inundation areas are shown in (**d**–**f**). Land areas exposed to erosion are shown in (**g**–**i**); insets show acceleration in areas exposed to erosion, when plotted versus time. The cumulative area (**j**–**l**) is the area exposed to one or more hazards.

To investigate the spatial extent to which sea level-related processes impact coastal lands, for each SLR scenario, we calculated the total land area exposed to each modeled hazard, as well as the cumulative land area exposed to one or more hazards (Fig. 3, left column). We also calculated the change in exposed area, relative to the baseline “present-day” area (future area - baseline area) (Fig. 3, middle column); and the areas normalized by the baseline area (future area/baseline area) (Fig. 3, right column).

For passive flooding at high tide (Fig. 3a–c), marine overland flooding (dark gray, single-hatching) is more prevalent than groundwater/storm drain flooding (white, cross-hatching). On Kaua‘i, exposure to groundwater flooding/drainage backflow from the ocean is projected to decrease between 0.60 m and 0.98 m of SLR because, in some areas, flooding that was previously related to groundwater/drainage, transitions to marine overland flooding as SLR increases. Results (Fig. 3b) suggest that 0.98 m of SLR will lead to an additional 27.8 ± 5.9 km^2^ of land exposed to passive flooding (both marine overland and groundwater/storm drain) on Kaua‘i (11.3 ± 3.3 km^2^), O‘ahu (13.3 ± 2.4 km^2^), and Maui (3.2 ± 0.5 km^2^), combined (± values are standard errors). Normalized areas show that, at 0.98 m SLR, land area exposed to passive flooding will be 4.2 times present-day exposure on Kaua‘i; 3.7 times on O‘ahu; and 2.6 times on Maui (Fig. 3c).

Land area exposed to wave inundation indicates that O‘ahu experiences the largest increase in exposure to wave inundation, at a growth rate that substantially exceeds the other two islands (Fig. 3d,e). Normalized inundation areas (Fig. 3f), however, indicate that Maui and O‘ahu will see the largest increase in land area exposed to waves, proportional to present-day exposure; and the two islands have similar normalized growth rates over time. While the exact mechanisms for the differences between islands require more study, it is possible that O‘ahu and Maui suffer from lower dune elevations due to: (1) flattened dunes on O‘ahu resulting from high coastal urbanization, and (2) less dune-building wind and wave energy on Maui, where most beaches are located on the leeward side of the island (less persistent winds) and in the shadow of close-by islands that buffer incoming wave energy. Results also indicate that, for the 0.98 m SLR scenario, an additional 14.0 ± 3.5 km^2^ of land is exposed to wave events on Kaua‘i (3.5 ± 0.9 km^2^), O‘ahu (7.9 ± 2.0 km^2^), and Maui (2.7 ± 0.8 km^2^), combined (Fig. 3e). This translates to 3.1, 3.0, and 1.9 times present-day exposure on Maui, O‘ahu, and Kaua‘i, respectively, for the 0.98 m SLR scenario.

Land area exposed to erosion is projected to increase by roughly the same amount over time on each island (Fig. 3g,h). At 0.98 m of SLR, an additional 7.9 ± 3.7 km^2^ of land is exposed to erosion on the islands of Kaua‘i (2.9 ± 1.4 km^2^), O‘ahu (2.7 ± 1.4 km^2^), and Maui (2.4 ± 1.0 km^2^), combined. Normalized exposure areas (Fig. 3i) indicate that Maui will see the largest increase in land area exposed to erosion, proportional to the present-day area, however, errors are large. At 0.98 m of SLR, projected land exposure is 2.4 times the present-day exposure on Maui; Kaua‘i and O‘ahu can expect land exposure areas that are 1.8 and 1.9 times the present-day area, respectively. As mentioned above, the erosion model differs from the passive and wave models, in that it depends on the elapsed time to reach each future sea level, rather than just the future SLR values themselves. In the erosion model, time and SLR are linked by the IPCC RCP 8.5 projection (Table 1), which indicates acceleration in SLR with respect to time. Thus, the elapsed time between equal increments of SLR reduces as sea level rises. For example, a 0.1 m increase in sea level (from 0.1 to 0.2) corresponds to an 18.4 year time interval in the IPCC projection; while a 0.1 m increase in sea level (from 0.5 to 0.6) corresponds to a 9.5 year time interval. Hence, for incremental increases in sea level, the term in Eq. (1) that includes the long-term trend (r) actually reduces in magnitude at higher sea levels. In this study, the apparent deceleration in erosion area growth, with respect to SLR, occurs because, overall, the reduction in the linear term (trend) is greater than the increase in the SLR term (right side of Eq. 3) as sea level rises. When plotted against time, however, exposure to erosion appears to accelerate (inset, Fig. 3h,i).

Cumulative exposure areas (Fig. 3j,k) indicate an additional 41.8 ± 14.3 km^2^ of land exposed to one or more hazard for the 0.98 m SLR scenario for the islands of Kaua‘i (15.4 ± 5.6 km^2^), O‘ahu (20.1 ± 5.7 km^2^), and Maui (6.3 ± 2.9 km^2^), combined. Normalized cumulative exposure areas (Fig. 3h) are essentially equivalent between the islands, for all modeled SLR values, suggesting land area which is roughly triple the present-day cumulative exposure on each island, at 0.98 m of SLR. There is no obvious reason for the equivalence.

### Land exposure growth rates with respect to SLR

**Figure 4:**
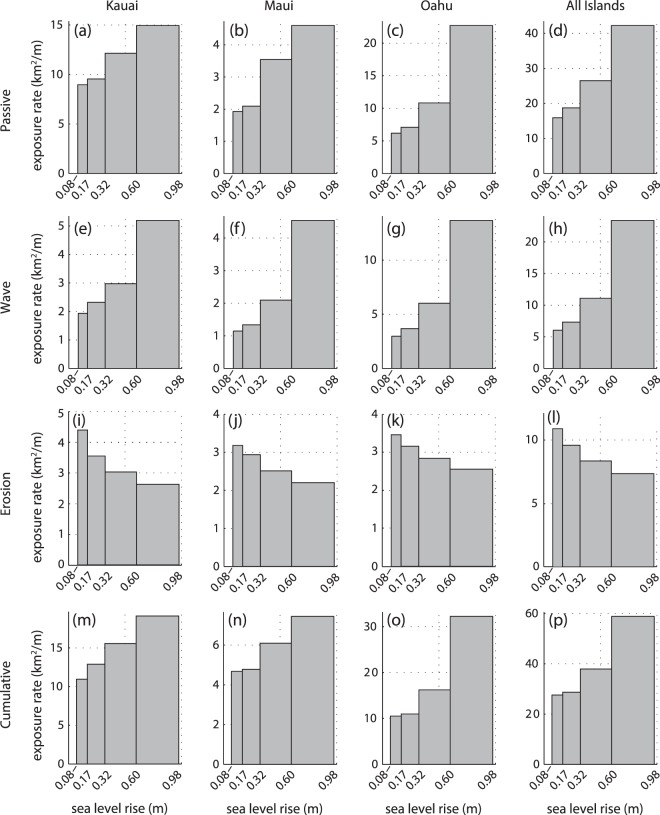
The simple rate at which new land becomes exposed to each SLR hazard, per unit SLR, is shown for the four intervals of higher sea level: 0.08–0.17 m, 0.17–0.32 m, 0.32–0.60 m, and 0.60–0.98 m. (**a**–**d**) Passive flooding exposure rates (includes both marine overland flooding, and groundwater/storm drain flooding). (**e**–**h**) Wave inundation exposure rates. (**i**–**l**) Erosion exposure rates. (**m**–**p**) Cumulative exposure rates.

To investigate how the extent of impacted lands changes, as a consequence of SLR, we calculate a simple rate at which lands become exposed to sea level-related hazards, with respect to SLR, by taking the difference in consecutive exposed areas and dividing that value by the difference in consecutive sea levels (Fig. 4). Aside from erosion, higher intervals of SLR are related to greater expansion rates of the exposure area, per unit of SLR. For example, in the case of passive flooding (which includes both marine overland flooding and groundwater/storm drain flooding) on O‘ahu (Fig. 4c), when sea level is between 0.32–0.60 m above present MSL, the area exposed to flooding expands at a rate of ~11 km^2^ per m of SLR (or ~1 km^2^ of newly flooded land for each 10 cm of SLR); when sea level is between 0.60–0.98 m above present MSL, the area exposed to flooding expands at a rate of ~23 km^2^ per m of SLR (or ~2 km^2^ of newly flooded land for each 10 cm of SLR). The only hazard that indicates a reduction in expansion rate at higher SLR intervals is erosion (Fig. 4i–l); note that the rates are still positive, however, which means that SLR continues to expose more land to erosion, but at reduced rates as sea level rises. Overall, the projected rate of new land area that will become exposed to one or more hazards over all three islands (Fig. 4p) roughly doubles: from ~30 km^2^ per m of SLR (for sea level between 0.17–0.32 m above present MSL), to ~60 km^2^ per m of SLR (for sea level between 0.60–0.98 m above present MSL).

### Contribution of individual hazards to the cumulative area

**Figure 5:**
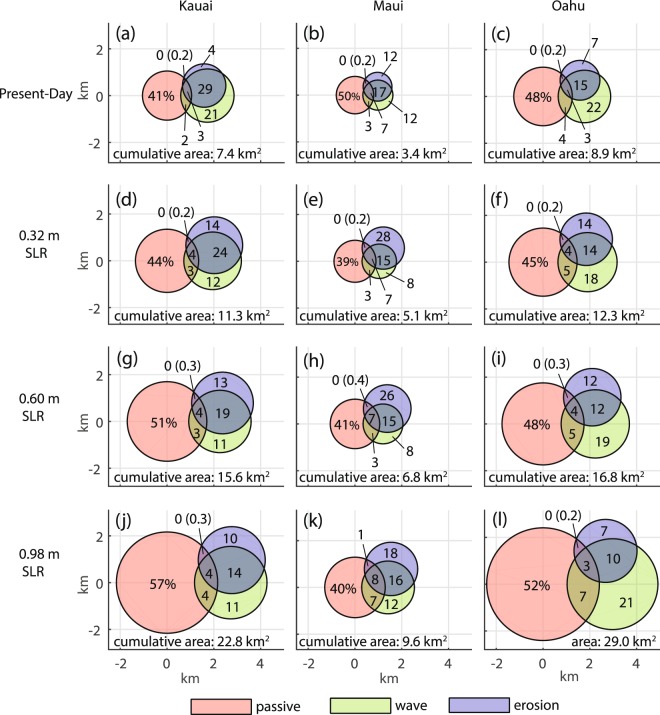
The relation between area exposed to passive flooding (red; includes both marine overland flooding, and groundwater/storm drain flooding), wave inundation (blue), and erosion (green) is shown as Venn diagrams. Each intersecting region represents the land area exposed to the corresponding combination of hazards. The percent of land area represented by each region is displayed in the diagram (the precision to one decimal is also shown in parenthesis, for percentages that round to zero). The land area that is exposed to one or more hazards (cumulative area, or union between all land areas) is given at the bottom of each diagram. Diagrams are shown for the (**a**–**c**) present-day scenario, (**d**–**f**) 0.32 m, (**g**–**i**) 0.60 m, and (**j**–**l**) 0.98 m SLR scenarios.

To quantify the effect of augmenting the passive flood model with the wave and erosion models, we calculate the areas exposed to combinations of overlapping hazards and compare them to the cumulative exposure area. Figure 5 displays Venn diagrams, in which areas exposed to each hazard type are depicted as circles (passive = red; wave = green; erosion = blue). For clarity, we do not distinguish between the two passive flooding types (marine overland; and groundwater/storm drain backflow from the ocean). Wave inundation and erosion show the largest overlap between modeling components. This is consistent with the nature of these processes: wave inundation and erosion first impact the shoreline, then travel inland. By contrast, passive flooding occurs both near the shoreline and in low-lying inland areas. Recall that, our method of mapping land exposure to wave inundation includes removing any static (zero velocity) inland water. Thus, wave and passive flooding only intersect near the coast. Despite the overlap between wave inundation and erosion, 35–54% of the cumulative area in each diagram is represented by modeling these two processes (not including any overlap with passive flooding), which is the area that would be missed by performing only the passive flood model. We do not present the diagram for the 0.16 m SLR scenario because of its similarity to the 0.32 m SLR scenario.

## Discussion

Our results indicate that there is considerably more land at risk of flooding or erosion, by SLR, than shown by simple passive flood mapping alone. In Hawai‘i, coastal planning responses must account for wave inundation and erosion to avoid missing 23.3 km (38%) of threatened land over all three islands, under 0.98 m of SLR; this is equivalent to nearly four times the land area of existing beaches. Such an oversight can have significant adverse consequences for coastal communities. And, while the percent contribution of erosion alone to cumulative SLR risk appears small under 0.98 m SLR (10%, 18%, and 7% on Kaua‘i, Maui, and O‘ahu, respectively; Fig. 5j–l), the impact is likely considerable, given the prolific coastal development along Hawai‘i’s shorelines (e.g. Waikīkī on O‘ahu; Fig. 1d), which shows no sign of lessening in coming years. On undeveloped coasts, erosion can lead to total loss of a beach when there are limited sand deposits inland of the existing beach, such as on the north shore of Maui, where clay deposits back narrow beaches. Thus, relying only on the passive flood model will likely under-estimate these impacts, resulting in insufficient planning.

SLR clearly leads to increased land exposure to hazards (Fig. 3). Yet, the amount of exposure is not linearly-related (or proportional) to the amount of SLR (Fig. 4). For example, the extent of land exposed to passive flooding (marine overland + groundwater/storm drain backflow) on O‘ahu (Fig. 4c), per unit SLR, grows at an accelerated rate, and shows a relatively sizable rate increase when SLR exceeds 0.60 m. This sharp increase indicates that there is a critical elevation, near 0.60, above which SLR leads to rapid expansion in land area exposed to passive flooding on O‘ahu. By contrast, the largest acceleration in passive flooding on Maui (Fig. 4b) occurs when SLR exceeds 0.32 m, indicating that, in general, Maui has a lower critical elevation for passive flooding (~0.32 m) than O‘ahu (~0.60 m). This is consistent with Kanē *et al*.^52^, which determined critical elevations separating phases of slow and fast passive flooding for one wetland on O‘ahu (critical elevation = 0.6 m) and two wetlands on Maui (critical elevations = 0.2 and 0.6 m). The threshold between slow and fast phases is attributed to the prevalence of low-lying coastal plains that exist near the critical elevations. Similarly, the expansion rate of the land area exposed to wave inundation increases suddenly when SLR exceeds 0.60 m on all islands (Fig. 4e–h), indicating a critical elevation near 0.60 m, above which SLR leads to rapid expansion in land area exposed to wave inundation. Since the amount of land exposed to passive flooding and wave inundation only depends on the future sea level, and not the year at which that level is expected to be reached, the accelerated rates of land area exposed to passive flooding and wave inundation are a consequence of the local shape of the land, rather than the acceleration of SLR over time.

This conjecture does not hold for the erosion model, because, as discussed above, erosion risks depend on the relationship between time and SLR. The apparent reduction in the rate (Fig. 4i–l) of new land exposed to erosion per unit SLR, when plotted against sea level, is at least partially a consequence of the acceleration in the IPCC SLR curve, coupled with a linear (in time) term for sediment transport. As a function of time, however, there is an apparent acceleration of land area exposed to erosion, as gleaned from the insets in Fig. 3e,f. Nonetheless, the simple exposure rate for erosion suggests that, as sea level rises, erosion is not as susceptible as the other hazards are to extreme and sudden increases in exposed land area. Yet, it is possible that the simple model that we use does not capture the full extent of future shoreline change because it assumes that sediment transport rates remain constant over time.

In addition to SLR leading to increased land exposure to hazards; once a piece of land is exposed to a hazard, the magnitude of impact, or the degree to which that hazard negatively impacts that particular parcel, also increases with SLR, even if the extent of land exposure stays the same. For example, it is a reasonable assumption that once a piece of land becomes exposed to passive flooding, it will experience higher flood depths with higher sea levels. Our modeling indicates that wave inundation poses a similar problem. Bivariate (depth, velocity) histograms of depth-velocity pairs (Supplementary Fig. S1) show a higher occurrence of larger-magnitude depth-velocity pairs as sea level rises. This suggests that, as sea level rises, any particular unit of land area will likely be subject to higher water levels moving at faster speeds, as a result of wave inundation. For erosion, although the exposure area does not increase at an accelerated rate with respect to SLR, the rate of shoreline retreat is expected to accelerate *over time* (assuming future acceleration in SLR): from the mean historical shoreline change rate of −0.08 ± 0.02 m/yr to a mean rate of −0.32 ± 0.03 m/yr by the year 2100 (±values are the 95% confidence interval for the mean; negative values indicate retreat; from Supplementary Table S2). Overall, future SLR will likely lead to an increase in the magnitude of impact that each hazard has on coastal land (for erosion, this assumes that SLR accelerates in the future), which, in turn, leads to greater potential for the hazard to be damaging. Flood damage to buildings is commonly projected by applying a depth-damage function^53–55^, where higher flood depths correspond to higher percentages of damage to buildings and their contents. Thus, once a land area is exposed to any of the hazards that we modeled, results suggest that hazards will become increasingly more damaging as sea level rises.

The specific risks associated with SLR vary between locations, and fully understanding these can be hampered by lack of data and funding. The physical processes that we modeled are more appropriate for use in oceanic islands. However, small island nations, especially in the Pacific, often lack the required data for these models (e.g., topo/bathy LIDAR, wave time series, historical rates of shoreline change, etc.), and lack the funds to conduct such modeling or collect the needed data. For those locations, data acquisition is the primary need. In the interim, we recommend using any combination of available forecasting methods in planning for SLR, as some guidance is still better than none, and will raise awareness that SLR is a threat, and that more data is needed.

There are limitations to our approach and the models that we used. Projections for future SLR include large uncertainty values, mainly because of limited understanding of ice sheet contributions to the sea level budget, and unknowns related to curbing greenhouse gas emissions. Large uncertainty in the SLR projections contributes to large uncertainty in the erosion model. Concerning wave inundation, some physical systems might require at least a 2-dimensional (2D) model (as opposed to the 1D model we used) to capture alongshore wave transformation more accurately. Moreover, waves are run on a DEM which was not updated to reflect future changes in beach morphology. Thus, when creating adaptation strategies, it would be prudent to perform higher-resolution investigations that use our modeling results to focus the scope of such studies. For example, 2D wave modeling might provide required accuracy for planning where the reef is highly irregular. The need for additional study depends on individual sites and project objectives.

## Conclusions

We found that modeling the impacts of the annual high wave event and coastal erosion, in addition to the standard passive (bathtub) flood model, is essential to providing a more comprehensive vulnerability assessment of future SLR scenarios in Hawai‘i. Depending on the island and SLR scenario, our results show that relying solely on the passive model results in missing 35–54 percent of the total land area that is exposed to one or more hazards.

The geographic extent and magnitude of SLR impacts are also expected to increase rapidly with SLR. The rate at which new land becomes exposed to one or more hazards, per unit SLR, nearly doubles from ~30 km^2^ per m of SLR (for scenarios between 0.17–0.32 m) to ~60 km^2^ per m of SLR (for scenarios between 0.60–0.98 m). This increase is mainly a consequence of prevalent low-lying coastal plains. For Hawai‘i, we identified the following critical elevation thresholds, beyond which damage accumulates rapidly: 0.32 and 0.60 m for passive flooding on Maui and O‘ahu, respectively; and 0.60 m for wave flooding on all islands. Once lands are exposed to hazards, they are subsequently subjected to increasing flood depths and velocities as sea level rises, which leads to greater damage to structures and habitats that exist within threatened areas.

Because of the rapid increase in (1) land exposure to SLR hazards and (2) magnitude of SLR impacts, the time to engage in long-term planning is now. Taking this next step will allow for the creation and implementation of adaptation measures specifically-tailored to meet each locality’s unique SLR vulnerability profile. With sufficient modeling to identify potential hazard vulnerabilities, individual locations become well informed to craft adequate SLR adaptation responses. As our study shows, it is crucial that SLR impact modeling is conducted at the local scale and include as many hazards as possible for the sake of building more resilient communities.

## Methods

### Modeling passive marine and groundwater flooding at high tide

Passive flood modeling is described in the main text, under the section: Approach and Models, Passive flooding at high tide. For validation, modeled flooded areas were compared against known elevations during present day conditions.

In passive modeling, we assume that the amount of SLR is known. Thus, we estimate the error in the baseline scenario ${\sigma }_{base}$ as the increase in exposed land area that corresponds to ~10 cm of SLR (10 cm is the vertical error in the DEM described below):

2 $${\sigma }_{base}=Are{a}_{SLR=0.18m}-Are{a}_{base,(SLR=0.08m)}$$

For higher SLR values, we assume that the error increases proportionally to projected exposure area; and, the constant of proportionality is the fractional uncertainty (error in area divided by estimated area) of the baseline area. This helps account for larger errors that correspond to larger increases in exposure area when, at higher SLR scenarios, ground slopes are generally milder than the baseline scenario. At milder slopes, the vertical error in the DEM leads to larger errors in exposed area projections. The resulting error for projected exposure under future SLR is:

3 $${\sigma }_{future}=Are{a}_{future}\cdot \frac{{\sigma }_{base}}{Are{a}_{base}}$$

### Digital Elevation Model for Passive Flood Modeling

The DEM for passive flood modeling is a digital surface that represents the local terrain. It consists mainly of a 1 m resolution bare-earth DEM that was derived from light detection and ranging (LiDAR) data collected in 2013 by the U.S. Army Corps of Engineers (USACE) (http://shoals.sam.usace.army.mil/). This DEM extends seaward to depths of approximately 35 m, while the landward coverage extent is typically between 400 and 1000 m inland from the shore. The 2013 DEM was augmented with the 3 m resolution NOAA SLR Viewer DEM^36^ to fill in low elevations that extend far inland. Only the terrain portion of the DEM (not the bathymetric portion) was used for passive flood modeling.

Due to the collection and processing methods used on the 2013 USACE LiDAR data, many streams and wetlands are poorly classified or characterized. Where possible, satellite imagery of a similar date was used in conjunction with a hillshade rendering of the DEM to identify misclassified or misinterpreted areas and apply a corrected elevation to the depicted stream or wetland area that approximates nearby valid elevations. This “hydro-flattening” is intended to better depict low-lying wetland areas and streams in the modeling, and to ensure waterway connectivity to the ocean in the presence of bridges and similar structures.

### Modeling wave inundation

The non-hydrostatic mode of XBeach was used in this study to capture short wave contributions to water levels, which are often required to accurately model wave overtopping^56^. For validation in Hawai‘i, we modeled wave heights and water levels using XBeach, and compared them against measurements from an array of bottom-mounted pressure sensors located on O‘ahu.

Projections of water depths and velocities were estimated, for each SLR scenario, as follows. We run a 1-hour (model time) XBeach simulation using maximum annually recurring wave conditions along shore-normal profiles spaced 20 m apart. Then, we define the extreme water level as the average of the five highest water elevations at each grid point along each profile, and identify the corresponding velocity. Water depth is calculated as the difference between the annual extreme water level surface and the ground elevation. Depths and velocities are then interpolated to create 2D surfaces using ArcGIS, and resampled at 5 m grid spacing. Modeled parameters (depth, velocity) at grid locations with velocity <0.1 m/s or depth <0.1 m are omitted to ignore static, inland water that does not result from waves, and to remove thin veneers of water excursion. The error in land area exposed to wave inundation is determined using the same method described in modeling passive flooding, except the ${\sigma }_{direct}$ value is calculated using a 17 cm vertical error, rather than 10 cm, to account for additional uncertainty in the wave model.

### Data and boundary conditions

We augmented the 1 m resolution 2013 USACE DEM with lower resolution (10–50 m) bathymetric data collected by the Hawai‘i Mapping Research Group for water depths that exceed the maximum LiDAR depth range (roughly >35 m depth). However, the lower resolution data is rarely used since most modeling is conducted over shallower water depths. To fill bathymetry gaps within nearshore areas, we use a 10 m DEM that was created from all 1999–2003 USACE bathymetric and topographic surveys available from NOAA’s Digital Coast^36^. Data gaps in shallower water (<10 m) are left unfilled because we find that these gaps only occur in small patches whose spatial footprints are much smaller than the resolution of the 1999–2003 10 m DEM; and using the lower resolution DEM values to fill these gaps results in obvious elevation errors near the shoreline. Instead, we linearly interpolate across any small gaps in elevation after we have extracted the 1D profiles from the DEM.

Mean water levels are determined by increasing the water level above MHHW by the desired amount of SLR. To obtain wave information at the local level, we use the wave forecast available from PacIOOS^57^, which has been providing a 5-day hourly forecast that is calibrated using local wave buoys, for Hawaiian regions since 2009. This forecast is produced by using the WaveWatchIII and SWAN wave models to dynamically downscale atmospheric forcing from the NCEP Global Forecast System weather model^57^. From this forecast, we use the hourly time series of significant wave height, peak wave period, and dominant direction, for the years 2009–2015.

We then follow the method presented by Vitousek and Fletcher^39^ and extract peak wave heights from the time series of significant wave height, and fit those to a generalized extreme value (GEV) distribution, from which the maximum annually occurring significant wave height and the associated peak period are found. To determine the dominant direction, we perform the GEV fit on subsets of wave series that are limited to incoming wave directions within 30-degree bins, and let the bin ranges shift by 15 degree increments (a maximum of 24 bins; there will be overlap between bins). The direction from which waves will produce the highest run-up is typically shore-normal, and has the largest wave height and period. On the rare occasion that these three conditions do not align, we use expert knowledge and generally select the direction corresponding to longer period waves. See Supplementary Tables S2–S4 (excel spreadsheets) for wave parameters used.

We repeat this procedure on wave data at locations spaced 1.5–2.5 km apart, in roughly 25–35 m depth around each island. The annually-recurring maximum wave parameters are then interpolated along the offshore boundary to match profile locations. Since projections of future wave conditions due to climate change do not indicate significant changes in wave characteristics near Hawai‘i, we use the same wave parameters for all future SLR scenarios^58^. From the wave parameters, we use XBeach routines to generate the 1-hour time series of incoming waves.

Friction due to bottom roughness affects wave flow velocity. To determine bottom roughness, we use two mapping products (GIS polygon layers) that classify the geology and structural type of land and sea topography. For land areas, we use the USGS geology classification map for Hawai‘i^59^; for submerged areas, we use benthic structural classification maps (e.g. sand, spur and groove reef, pavement reef) produced as part of the Mapping of Benthic Habitats for the Main Eight Hawaiian Islands project for NOAA’s National Ocean Service Center for Coastal Monitoring and Assessment^60^. We then assign friction values to each geologic type and structural classification (see Supplemental Tables S5–S6 (excel spreadsheets) for friction values). Then, via a lookup table, we assign these values to each polygon. The polygon layers are merged and gridded to match the DEM grid size. Because rough reef platforms can episodically become exposed and/or covered by sand, and because the USGS geology maps are lower-resolution than our DEMs, we edited some areas of the USGS and NOAA maps to match conditions at the time of the DEM data collection. When needed, we hand-digitized replacement polygons that more accurately represent structural classification by looking at the existing DEM and most-recent aerial photo, then merging this new layer with the existing one.

### Modeling chronic coastal erosion

Following the probabilistic method described in Anderson *et al*.^17^ (and introduced in equation (1) above) we produce a probability density function (pdf) for the projected vegetation line *y*_*veg*_(*t*) by numerically combining the pdfs for each term on the right hand side of equation (1). We then determine the erosion hazard line as the 80th percentile,${y}_{veg,80 \% }={{F}_{veg}}^{-1}(80/100)$ where ${F}_{veg}={\int }_{-\infty }^{80/100}pd{f}_{veg}$ is the cumulative distribution function of the pdf for the projected vegetation line. The 80th percentile is used to align the erosion hazard estimate with the other models (passive flood and wave) that both use the upper limit (83rd percentile) of the IPCC probability range for future sea level.

Calculations are performed at shore-normal transects, spaced 20 m apart along the shore. The erosion hazard line at each transect (the 80th percentile of the pdf for the projected vegetation line) is then projected back to map coordinates and exported as a GIS line shapefile. These calculations are done using Matlab. Some edits are manually performed in ArcGIS to smooth areas where transects overlap along coasts with high curvature. Erosion hazard areas are created in ArcGIS by connecting the erosion hazard line with the existing shoreline (MSL elevation contour).

The error in new land area exposed to erosion for future sea level *S*_*f*_ and time *t* is determined as ${\sigma }_{area({S}_{f},t)}=\sum _{i}{\rm{\Delta }}x\cdot {\sigma }_{i,({S}_{f},t)}$, where ${\sigma }_{i,({S}_{f},t)}$ is the standard deviation for the *i*^th^ projected shoreline position, and Δ*x* is the distance between transects (20 m).

### Data used in erosion modeling

We use historical shorelines and vegetation lines that were digitized as part of the USGS National Assessment of Shoreline Change^48^ and augmented with newer digitized shorelines for the north Maui and west Maui regions^49^. The positional error in each digitized shoreline was estimated as the root mean square of up to seven sources of positional error. The reader is referred to Fletcher *et al*.^48^ for a detailed description of the historical shorelines.

At some beaches, past human activity, such as sand mining in the early 20^th^ century, can influence the long-term shoreline change rate and violate the assumption that shoreline change remains fairly constant over the roughly 80 years of available data. To capture ongoing behavior of the beach, we calculate shoreline change rates only over the period of time following any significant beach alternation, such as sand mining. In the case where a beach is completely lost to erosion and a seawall has been built, we calculate the shoreline change rate up to and including the first shoreline where the wall was erected. Since we cannot predict if a wall will be built or removed, we use the preexisting rate (before the wall was built) to provide an estimate of exposure to erosion, in the beach’s natural state.

A straight line is fit to the historical shoreline data using weighted least squares regression^61,62^. Rates are then smoothed in the alongshore direction using a weighted [1 3 5 3 1] moving average to reduce spurious variations between adjacent beach locations.

To determine beach slope, we use the collection of biannual cross-shore beach surveys (1994–1999) on beaches of O‘ahu and Maui^63^, as well as additional surveys at 35 locations on O‘ahu and 27 locations on Kaua‘i over the period 2006–2008^17^. Following Anderson *et al*.^17^, we extract, for each repeated beach survey, the slope between two morphologic features: the beach toe^64^, which is located at base of the foreshore, and; either the point at which the repeated surveys converge (depth of closure) or the intersection of the sandy profile with the reef platform^65^. The final beach slope, tan*β*, is determined as the average of the individual slopes. We use the standard deviation of the slopes as our estimate for the error associated with tan*β*. If more than one profile location exists along a study area, slopes are interpolated using cubic splines.

## Electronic supplementary material

Supplementary Material

Dataset 1

Dataset 2

## Data Availability

The data used in this study are publicly available from the parties identified in the text or in the citations. Other intermediate products are available upon request to tranders@hawaii.edu.
