# Publisher Correction: Modeling multiple sea level rise stresses reveals up to twice the land at risk compared to strictly passive flooding methods

**DOI:** 10.1038/s41598-019-45275-z

**Published:** 2019-06-12

**Authors:** Tiffany R. Anderson, Charles H. Fletcher, Matthew M. Barbee, Bradley M. Romine, Sam Lemmo, Jade M. S. Delevaux

**Affiliations:** 1 University of Hawaiʻi at Mānoa, Department of Geology and Geophysics, School of Ocean and Earth Science and Technology, Honolulu, HI 96822 USA; 2 Geography Research Unit, University of Oulu, P.O. Box 8000, Oulu, 90014 Finland; 3 University of Hawaiʻi Sea Grant College Program, Honolulu, HI 96822 USA; 4 Hawaiʻi Department of Land and Natural Resources, Office of Conservation and Coastal Lands, Honolulu, HI 96813 USA

Correction to: *Scientific Reports* 10.1038/s41598-018-32658-x, published online 27 September 2018

The original HTML version of this Article contained a typographical error in the spelling of the author Jade M.S. Delevaux, which was incorrectly given as Jade M.S.M.S. Delevaux. This has now been corrected in the HTML version of the Article.

