# Supplementary material for: Modeling multiple sea level rise stresses reveals up to twice the land at risk compared to strictly passive flooding methods
Source: Sci Rep. 2018 Sep 27;8:14484. doi: 10.1038/s41598-018-32658-x (PMC6160426; doi:10.1038/s41598-018-32658-x)
Supplement: Supplementary file 1 — Supplementary Material [file 41598_2018_32658_MOESM1_ESM.docx]

Supplementary material for

“Modeling multiple sea level rise stresses reveals up to twice the land at risk compared to strictly passive flooding methods”

Tiffany R. Anderson^1,*^, Charles H. Fletcher^1^, Matthew M. Barbee^1,2^, Bradley M. Romine^3,4^,

Sam Lemmo^4^, and Jade M.S. Delevaux^1^

*^1^University of Hawai‘i at Mānoa, Department of Geology and Geophysics, School of Ocean and Earth Science and Technology, Honolulu, HI, 96822 USA*

*^2^University of Oulu, Geography Research Unit, Oulu, 90014 Finland*

*3University of Hawai‘i Sea Grant College Program, Honolulu, HI 96822 USA*

*^4^Hawaii Department of Land and Natural Resources, Office of Conservation and Coastal Lands, HI, 96813 USA*

**Corresponding author: tranders@hawaii.edu*

**Supplementary information on SLR scenarios**

We use the IPCC global mean SLR projections, rather than localized ones, because: (1) the islands in this study are either vertically stable (not subsiding or uplifting) or exhibit vertical motion that is negligible compared to errors in the digital elevation models (DEMs); and (2) local tide gauge data indicate that absolute SLR around Hawaii during the 20th century mimics the global mean^1^. However, recent research (since the IPCC report) suggests that the IPCC projections underestimate the true amounts of SLR for Hawaii^2–4^, so we use the upper limit of the “likely” range (83rd percentile) as the central tendency for future sea levels. The IPCC SLR projections are relative to 1986–2005. Similarly, all DEMs used in subsequent modeling are relative to mean sea level (MSL), which is based on the present National Tidal Datum Epoch (1983–2001).

**Supplementary information on technical differences between layers hosted online and those used in this study**

The GIS layers, created for display by TetraTech Inc., from the modeling described in this study (hosted online by PacIOOS; http://www.pacioos.hawaii.edu/shoreline/slr-hawaii/) differ from those used in all calculations and maps in this study because the two sets of GIS layers were produced independently. First, TetraTech, Inc. used a different (but similar) shoreline mask than we did (our study used the MSL contour derived from the 2013 LiDAR data) to clip the modeled areas that extend offshore, so the seaward boundary of exposure areas (i.e. shoreline) is different. Second, wave inundation layers used in our study exclude modeled pixels with very low velocity (<0.1 m/s) to further differentiate between dynamic wave processes and passive inland flooding; in the online version, these pixels are left in.


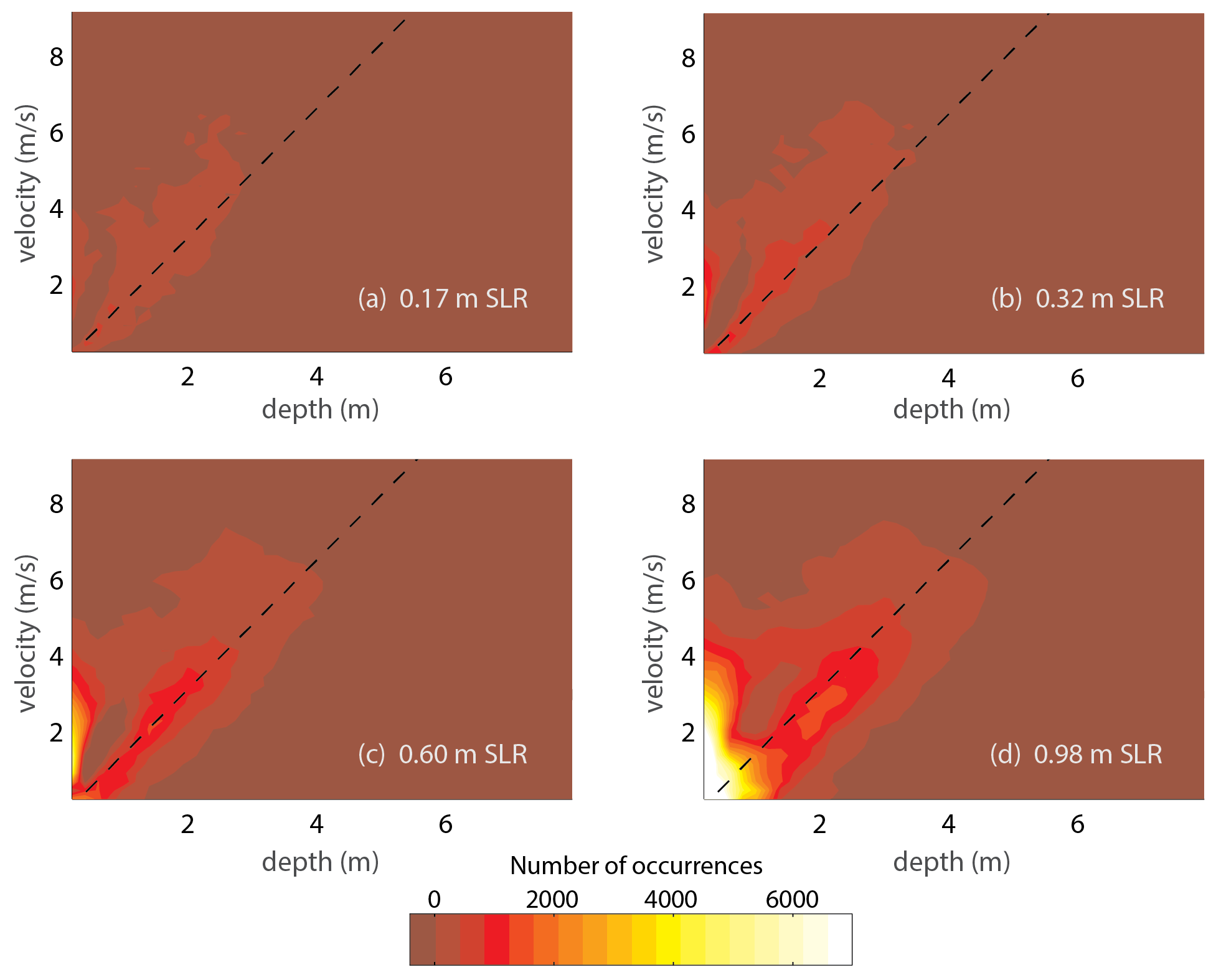


**Supplementary Figure S1.** The bivariate histograms show the number of occurrences, in excess of the baseline scenario, of 5 × 5 m grid cells (land area) that are subject to wave-induced flooding for each depth-velocity pair. The histogram shows that more land area is exposed to wave flooding at higher depths and velocities. The increase in flood occurrences for depth-velocity pairs near the dashed line suggests that wave impacts tend to increase at a depth to velocity ratio of 1 m to 1.7 m/s. The histogram also shows an increase in low depth (<0.5 m) and higher velocity (~1–4 m/s) impact over time, with an additional increase in low depth, low velocity occurrences in the 0.98 m scenario. Depth and velocity bin sizes are 0.2 m and 0.2 m/s respectively. Colored contours are shown for each 430 increase in the number of occurrences.

**Supplementary Table S1.** Historical and future mean predicted shoreline change rates for each Hawaiian Island (negative rates indicate retreat). Uncertainty (unc.) is the 95% confidence interval for the mean.

| Island | Length of Shoreline (km) | Year | Mean Rate ± unc. (m/y) | % Retreating | % Advancing |
| --- | --- | --- | --- | --- | --- |
| Kauai | 74.0 | Historical | –0.10 ± 0.08 | 72 | 28 |
|  |  | 2030 | –0.19 ± 0.08 | 82 | 18 |
|  |  | 2050 | –0.24 ± 0.08 | 86 | 14 |
|  |  | 2075 | –0.29 ± 0.09 | 89 | 11 |
|  |  | 2100 | –0.35 ± 0.10 | 92 | 8 |
| Maui | 65.4 | Historical | –0.13 ± 0.02 | 80 | 20 |
|  |  | 2030 | –0.22 ± 0.02 | 89 | 11 |
|  |  | 2050 | –0.27 ± 0.02 | 91 | 9 |
|  |  | 2075 | –0.32 ± 0.03 | 94 | 6 |
|  |  | 2100 | –0.38 ± 0.03 | 95 | 5 |
| Oahu | 106.5 | Historical | –0.04 ± 0.01 | 59 | 41 |
|  |  | 2030 | –0.12 ± 0.01 | 74 | 26 |
|  |  | 2050 | –0.16 ± 0.01 | 79 | 21 |
|  |  | 2075 | –0.21 ± 0.01 | 85 | 15 |
|  |  | 2100 | –0.27 ± 0.02 | 88 | 12 |
| All Islands | 245.8 | Historical | -0.08 ± 0.02 | 68 | 32 |
|  |  | 2030 | -0.17 ± 0.02 | 80 | 20 |
|  |  | 2050 | -0.21 ± 0.03 | 84 | 16 |
|  |  | 2075 | -0.27 ± 0.03 | 88 | 12 |
|  |  | 2100 | -0.32 ± 0.03 | 91 | 9 |

**Supplementary Tables S2-S4.** Maximum annually recurring wave parameters used in wave modeling are located in spreadsheet “Max_annual_wave_parameters_FINAL.xlsx”.

**Supplementary Tables S5-S6.** Friction values used in wave modeling are located in spreadsheet “Chezy_lookup.xlsx”.

**References**

1. Moore, J. G. Relationship between subsidence and volcanic load, Hawaii. *Bull. Volcanol.* **34,** 562–576 (1970).

2. Spada, G., Bamber, J. L. & Hurkmans, R. T. W. L. The gravitationally consistent sea‐level fingerprint of future terrestrial ice loss. *Geophys. Res. Lett.* **40,** 482–486 (2013).

3. Kopp, R. E. *et al.* Evolving Understanding of Antarctic Ice-Sheet Physics and Ambiguity in Probabilistic Sea-Level Projections. *Earths Future* (2017).

4. Sweet, W. V. *et al.* *Global and regional sea level rise scenarios for the United States*. 56p. (NOAA, 2017).
